# Differences in Abundances of Cell-Signalling Proteins in Blood Reveal Novel Biomarkers for Early Detection Of Clinical Alzheimer's Disease

**DOI:** 10.1371/journal.pone.0017481

**Published:** 2011-03-24

**Authors:** Mateus Rocha de Paula, Martín Gómez Ravetti, Regina Berretta, Pablo Moscato

**Affiliations:** 1 Centre for Bioinformatics, Biomarker Discovery & Information-Based Medicine, The University of Newcastle, Callaghan, Australia; 2 Departamento de Engenharia de Produção, Universidade Federal de Minas Gerais (UFMG), Belo Horizonte, Brazil; Mental Health Research Institute of Victoria, Australia

## Abstract

**Background:**

In November 2007 a study published in Nature Medicine proposed a simple test based on the abundance of 18 proteins in blood to predict the onset of clinical symptoms of Alzheimer's Disease (AD) two to six years before these symptoms manifest. Later, another study, published in PLoS ONE, showed that only five proteins (IL-1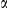, IL-3, EGF, TNF-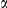 and G-CSF) have overall better prediction accuracy. These classifiers are based on the abundance of 120 proteins. Such values were standardised by a Z-score transformation, which means that their values are relative to the average of all others.

**Methodology:**

The original datasets from the Nature Medicine paper are further studied using methods from combinatorial optimisation and Information Theory. We expand the original dataset by also including all pair-wise differences of z-score values of the original dataset (“metafeatures”). Using an exact algorithm to solve the resulting 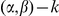 Feature Set problem, used to tackle the feature selection problem, we found signatures that contain either only features, metafeatures or both, and evaluated their predictive performance on the independent test set.

**Conclusions:**

It was possible to show that a specific pattern of cell signalling imbalance in blood plasma has valuable information to distinguish between NDC and AD samples. The obtained signatures were able to predict AD in patients that already had a Mild Cognitive Impairment (MCI) with up to 84% of sensitivity, while maintaining also a strong prediction accuracy of 90% on a independent dataset with Non Demented Controls (NDC) and AD samples. The novel biomarkers uncovered with this method now confirms ANG-2, IL-11, PDGF-BB, CCL15/MIP-1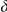; and supports the joint measurement of other signalling proteins not previously discussed: GM-CSF, NT-3, IGFBP-2 and VEGF-B.

## Introduction

In November 2007, a study published in Nature Medicine [1] immediately attracted both scientific and media attention. A multidisciplinary team led by Stanford researchers proposed a simple test, based on the abundance of 18 plasma signalling proteins, for early detection of clinical Alzheimer's disease (AD).

They showed that a molecular signature can be used to predict the onset of clinical symptoms of AD as early as two to six years before these symptoms manifest. These initial findings have important consequences as the scientific and social significance of being able to predict the onset of AD before clinical symptoms appear is of unquestionable benefit. The relative simplicity of the proposed method and the quality of the execution of the study contributed to the immediate interest in the scientific community.

The basic experimental design was remarkably simple. Using the abundance of 120 signalling proteins on a training set of 83 archived plasma samples, Ray et al. [1] identified an 18-protein signature, a subset of the set of 120 signalling proteins they were measuring, which proved to be useful to predict clinical symptoms of AD. The signature was able to show an overall effectiveness of 91% and 81% for AD predictability on two separate test sets: one comparing patients who developed clinical AD with Non-Demented Controls (NDC), and another comparing patients with Mild Cognitive Impairment (MCI) that developed AD with those who did not. Predicting AD within patients with a MCI as early as possible is particularly important because during the observation period of memory testing, which can take up to several months, profound neuropathological damage may occur [2].

Soon after this discovery was published, and using the same datasets available on the public domain, Gómez Ravetti and Moscato [3] showed that the abundance of only five proteins was sufficient to obtain an even better total prediction accuracy. They used an integrative bioinformatics approach, based on the (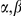)-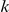-Feature Set methodology [4]–[12], that reduced the search of predictive biomarkers to only a subset of Ray et al.'s [1]: IL-1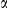 (interleukin 1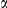), IL-3 (Interleukin 3 (colony-stimulating factor, multiple), EGF (epidermal growth factor (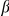-urogastrone)), TNF-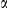 (Tumour Necrosis Factor 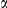) and G-CSF (colony stimulating factor 3 (granulocyte)).

Their results indicated that, using the abundance of just these five proteins together with simple established logistic-type classifiers, it was possible to distinguish NDC from samples with AD with a higher accuracy than that of the signature proposed by Ray et al. [1].

However, it is important to understand the context in which such accuracies are to be interpreted. As Ray et al. [1] already stated in their supplementary material, since many of the patients are still alive, it is not possible to be completely sure that the study participants that were labelled as AD samples are indeed individuals that will develop AD. An accurate AD diagnosis can only be obtained post-mortem with the histological analysis of brain material. The same can probably be stated of NDC, based on the same argument. Therefore, by “accuracy” what is actually reported in these performance tests is the *overall percentage agreement* with the current clinical diagnosis. That means that the existing classifiers have a high level of agreement with current clinical diagnosis but, as some of the samples might have been assigned an inaccurate label, they might also not be as robust as they could.

### Motivation

**Figure 1:**
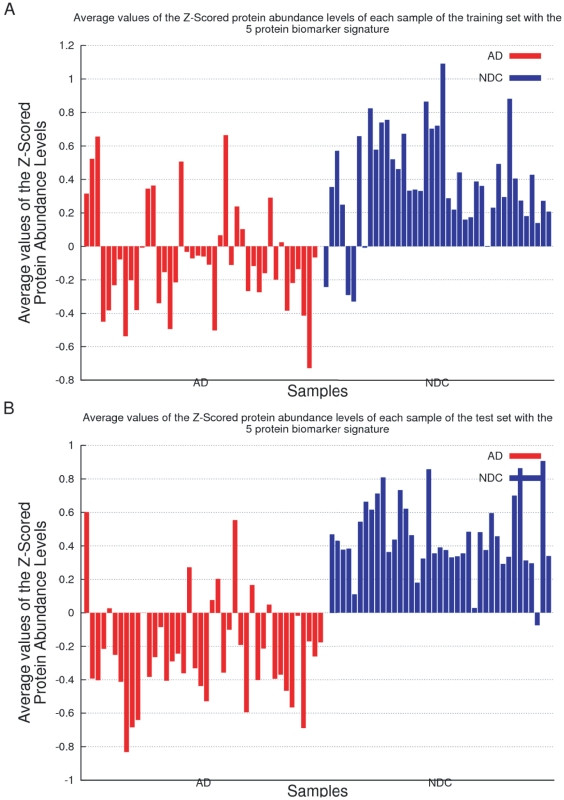
Stacked values of the Z-Scores of the 5-protein signature introduced by Gómez Ravetti and Moscato [**3**]. Figures 1A–B present the stacked values of the Z-Scores of samples in the training and independent test set respectively. The 5-protein signature includes the relative abundances of IL-1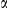 (interleukin 1 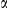), IL-3 (Interleukin 3 (colony-stimulating factor, multiple)), EGF (epidermal growth factor (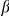-urogastrone)), TNF-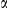 (Tumour Necrosis Factor 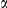) and G-CSF (colony stimulating factor 3 (granulocyte)) on a panel of 120 proteins used as reference set. Both Figures 1A–B shows that the stacked values of the Z-Scores of this panel of five proteins are lower in those patients that will develop clinical symptoms of AD in two to five years. The figures have samples as ordered in the original publication by Ray et al. [1]. In Figure 1A the leftmost 44 values correspond to those samples labelled ‘AD’, and in Figure 1B the leftmost 42 are labelled in the same way. The samples marked in red developed AD while the ones marked in blue are ‘Non-AD’ samples (Non-Demented Controls plus Other Dementias). Since a Z-Score transformation was performed on the dataset, the measured values of each protein are, in fact, relative to the variation of the other 119.

One of the most relevant characteristics of Gómez Ravetti and Moscato's study [3] is that they report results of not one, but 24 different classifiers available in the Weka software package [13]. They proposed that the consensus of the prediction of different classifiers, inspired by different mathematical principles, would provide a more reliable prediction than the results of a single classifier. This allowed to establish the relevance of the 5-protein signature since it was able to distinguish between AD samples and NDC with a higher overall percent agreement with clinical diagnosis. Since this average was obtained from the results of 24 different types of classifiers, instead of a single one, the study provided strong evidence that the 5-protein signature is indeed a useful biomarker panel. Figure 1 illustrates the performance of the uncovered 5-protein signature.

**Figure 2:**
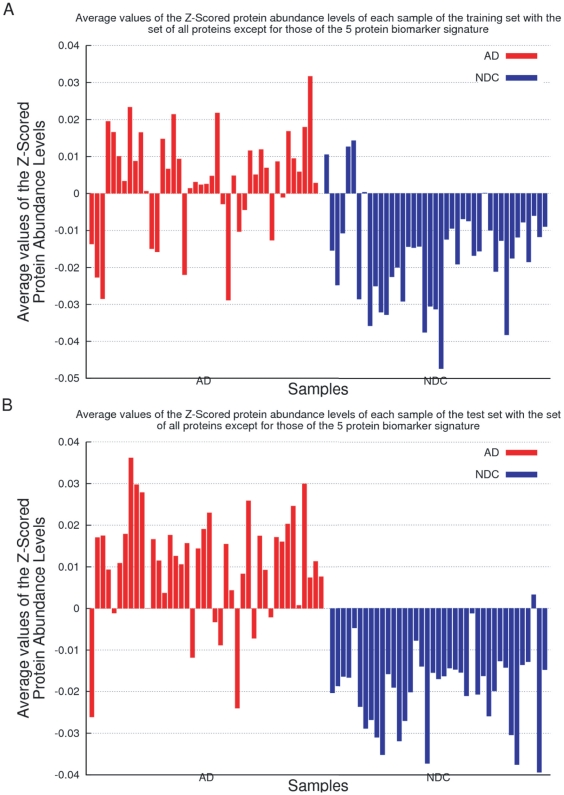
Stacked values of the Z-Scores of the set of all proteins except for those proposed by Gómez Ravetti and Moscato [**3**]. Figures 2A–B present the stacked values of the Z-Scores of samples in the training and independent test sets, respectively. The samples marked in red samples developed AD the ones marked in blue are ‘Non-AD’ samples (Non-Demented Controls plus Other Dementias). Like in Figure 1, since a Z-Score transformation was performed on the dataset, the measured values of each protein are, in fact, relative to the variation of the other 119. Because of that, and the fact that both Figures 1–2 distinguish well between classes, it is difficult to state whether the 5 proteins of Gómez Ravetti and Moscato [3] or the other 115 are the ones that are displaced with respect to the average.

Three facts are worth mentioning from the previous works by Gómez Ravetti and Moscato [3] and Ray et al. [1]: first, the majority of the classifiers performed better using the 5-protein signature than the 18-protein signature. Second, both in Ray et al.'s [1] and Gómez Ravetti and Moscato's [3] studies, some classifiers disagreed with the clinical diagnosis labels on the same samples of the datasets. Third, as Figure 1 clearly illustrates, the average of the Z-scores of their 5-protein biomarker is already a simple, yet powerful, discriminator between the two groups. However, all 5 proteins have, on average, a smaller Z-score in AD samples than in NDC. Since the measured protein abundances were standardised by a Z-score transformation, a positive value indicates the excess of a particular protein over the average value of 120 proteins. In essence, this means that the measures of each protein are, in fact, only relative to the variation of the other 119. Figure 2 shows that the average value of all proteins, excluding the 5 identified by Gómez Ravetti and Moscato's [3], also distinguishes well between the classes. Therefore, it is difficult to state whether the 5 proteins proposed by Gómez Ravetti and Moscato [3] or all other 115 are displaced with respect to the average.

Also, as both previous studies provide only aggregated results, this manuscript proposes a case-by-case analysis of the samples, with a methodology inspired by personalised medicine, using robust diagnostic methods. Although the overall performance of several classifiers is still reported in this work, the results under consideration are also systematically analysed for the samples individually.

Relative pair-wise protein variation of abundance levels are explored by expanding the original set of biomarkers with new “artificial” features, called “meta-features”, that model the relative protein imbalance. Using the meta-features, the relative proteins variations become explicit, providing useful information.

**Figure 3:**
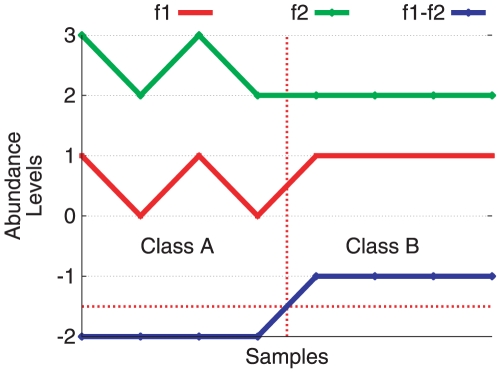
The difference of values of two features might be interesting to distinguish between two classes, even when those features alone are not useful for that purpose. In this example, the samples on the left hand side belong to Class A and the samples on the right hand side belong to Class B. The lines represent the Z-scored abundance levels of feature **f1,f2** and the meta-feature **f1-f2** for each sample. In this case, **f1** and **f2** are not effective at distinguishing between Class A and Class B, and would not pass the discretization algorithm's entropy filter [17]. However, if the difference between them is considered, we have a clear distinction, and the resulting meta-feature would be interesting and would pass the entropy filter. Roughly speaking, a feature is interesting to distinguish between two classes if it is possible to determine a pattern of up/down regulation of the samples' Z-scored abundance levels that characterizes each class uniquely (i.e.: in feature **‘f1-f2’**, all the samples Z-scored abundance levels are down regulated for Class A and up regulated for Class B. Such a distinction cannot be made either with features **‘f1’** or **‘f2’** alone.).

**Figure 4:**
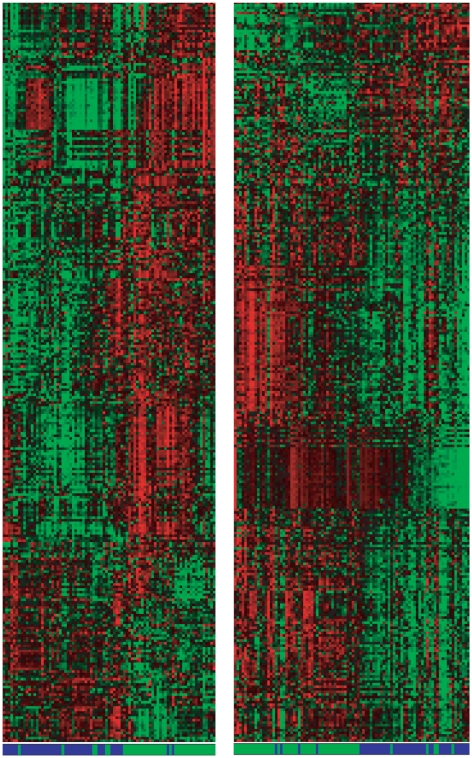
Heatmaps of the training (left) and test (right) sets, considering only the meta-features. The ordering of both rows and columns was done using the Memetic Algorithm presented in [15]. The Non-AD samples are marked in green, and the samples labelled AD are marked in blue. The ordering shows that there seems to be a robust molecular genetic signature that can be obtained by pattern recognition algorithms that explore all possible protein abundances differences in this panel of 120 proteins as variables of interest, a mechanism that quantifies the imbalance of cell signalling in plasma. An annotated version of this heatmap is available in the supplementary material. Please refer to it for detailed information about the ordering, selected features and values. On the training set, the samples that did not cluster with their associated clinical diagnosis group are samples s47, s77, s54, s50, s66, s68, s3 and s1, respectively.

As illustrated by Figure 3, since the difference of values between two features might be interesting to distinguish between two classes, even when those features are not useful for that purpose alone, the working hypothesis is that the use of meta-features might reveal if there exists a characteristic signature of the imbalance of cell signalling processes for AD prediction. Such a characteristic imbalance could also be regarded as a new molecular signature for predicting AD, which might add new information to other early detection tests or inspire entirely new ones. Indeed, the analysis of Figure 4 suggests that there is useful information within the meta-features that can distinguish between AD and NDC samples as most of the AD samples cluster together and only a few AD samples remain in the control group. The 290-protein Z-score differences presented in Figure 4 are a signature obtained with (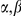)-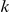-Feature Set methodological approach [3], [6]–[11], [14] on the set of meta-features only, and ordered using the Memetic Algorithm proposed by Moscato et al. [15]. The original 120 (single) features, which do not represent imbalance information, were not considered to guarantee that the discriminative information was indeed brought by imbalance information. These results motivate further study on the topic. The heatmap on the left of Figure 4 represents the samples from the training set, while the heatmap on the right represents the samples from the test set. The Non-AD (The test set samples include Other Dementia (OD) samples, which have not developed AD but are still demented controls) samples are marked in green, and the samples labelled AD are marked in blue.

## Materials and Methods

### Datasets

The modified datasets, used in the experiments, are based on those provided by the recent work of Ray et al. [1]. Quoting from their supplementary information: *“Autoradiographic films were scanned and digitized spots were quantified with the Imagene 6.0 data extraction software (BioDiscovery Inc.). Local background intensities were subtracted from each spot, and the average of the duplicate spots for each protein was normalized to the average of six positive controls on each membrane. For statistical analysis expression data from the two filters per sample were normalized to the median expression of all 120 proteins followed by Z score transformation (data file is available online).”*

score transformation has the effect of transforming the original distribution to one in which the mean becomes zero and the standard deviation becomes one. A Z-score quantifies the original score in terms of the number of standard deviations that the score is from the mean of the distribution. In other words, this means that a positive value in the original dataset indicates the excess of a particular protein over the average value of 120 proteins. That is, each value is relative to the variation of the other 119.

Equation 1 calculates the Z-score of the abundance level 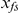 of protein 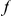 for a sample 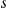, where 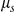 is the mean of the values of all features, for sample 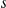, and 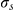 is the associated standard deviation.
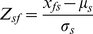
(1)


**Figure 5:**
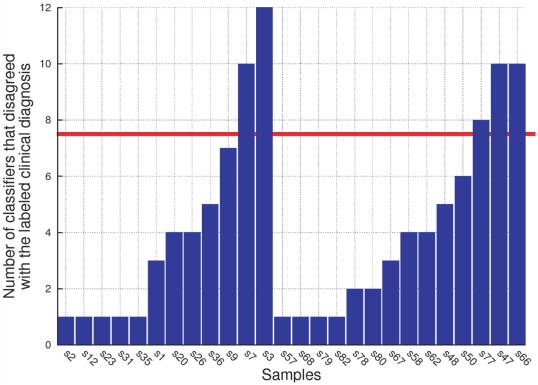
Histogram of the number of classifiers, out of the 25 under consideration, that disagreed with the clinical diagnosis attributed to the samples, when performing on the training set using the proposed 5 feature and 5 meta-feature signature. Samples with no such disagreement are omitted. Interestingly, many classifiers consistently disagree with the clinical diagnosis on the same samples, which hints for the questioning of their usefulness in the training set to distinguish between AD and NDC. One of the reasons for that is that the cell signalling could be altered by other medical conditions, such as other diseases and use of medication. Using an arbitrarily chosen threshold of 30% of the classifiers, or 8 classifiers or more (rounded up) disagreeing with the clinical diagnosis label of a sample, it is reasonable to suspect that samples s3, s7, s47, s66 and s77 are not suitable to be part of the training set. The signature used for this experiment includes the following features and meta-features: EGF, IL-1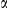, IL-3, TNF-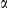, G-CSF, “BLC-RANTES”, “MIP-1d-IL-11”, “TNF-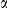 - ANG-2”, “TNF-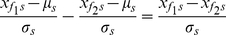 - FAS” and “IL-11-I-TAC”.

The original dataset consisted of a Training Set with 43 AD samples and 40 NDC samples, a Test Set with 42 AD samples and 39 NDC samples and another Test Set with 22 samples that had MCI and developed AD and 17 samples that had MCI but did not develop AD. The Other Dementia (OD) samples present in the original test sets are disconsidered for classification purposes, as they might have characteristics that could mask the pursued patterns. They are, however, still present on Figures 4 and 5. The OD samples found on the test set with NDC and AD samples have either Frontotemporal dementia or Corticobasal degeneration, while those found on the test set with samples that already had a MCI may have either a Lewy-Body dementia, Vascular dementia or Frontotemporal dementia.

The training and test sets considered in this paper are “enlarged” versions of the original. They include the original 120 features for each sample plus 7,140 “meta-features”, generated by applying the difference operator between each possible pair of features. Symmetric meta-features are not considered, as they are equivalent (e.g.: the information provided by a meta feature obtained by subtracting the value of two features **F1-F2** is equivalent to the one given by **F2-F1**).

As depicted by Equation 2, the meta-features model imbalance information, as each value is the displacement of one score with respect to the other involved in the meta-feature. Moreover, as illustrated by Figure 3, such a displacement may reveal interesting information to distinguish between classes that would not be obvious through the analysis of the features alone.
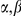
(2)


### Methods

The proposed computational methodology includes four basic steps on the expanded datasets, in this order: (1) feature selection, (2) classification, (3) analysis and (4) filtering.

Feature selection is performed using the same methodology presented in [3], [6]–[11], [14], [16]: first, the dataset is pre-filtered and discretised using Fayyad and Irani's [17] entropy-based algorithm, which minimizes the class entropy and discards features according to the Minimum Description Length principle. The result is an instance of the (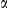)-k-Feature Set problem [3], [6]–[11], [14]. In this combinatorial optimization problem, three parameters are necessary: 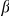, which determines the number of features that must explain the dichotomy between samples in different classes; 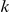, which determines how many features must explain the similarities between samples in the same class; and 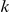, which specifies the size of the desired signature.

In this work, the five features of Gómez Ravetti and Moscato [3] are forced into the signature and 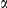 is set to 10, aiming to obtain a signature of about the same size as Ray et al.'s [1], while doubling Gómez Ravetti and Moscato's [3] and allowing the same number of new features or meta-features to be introduced. The rationale behind this is to guide the search towards a small signature with features and meta-features that are related to the ones that are already known to be effective in distinguishing between NDC and samples with AD, therefore helping to guide the search towards an even more effective signature. The 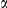 parameter is chosen to be the maximum possible such that the combinatorial optimization problem admits at least one feasible (i.e.: it is possible to explain the differences between the samples in different classes with at least 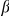 features, and the similarities between those in the same class with at least 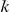 features, for all pairs of samples) solution, assuming a fixed signature size (a defined 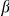) and not considering any restrictions imposed by a 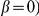 value (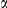. One way to determine this is to count the number of features that differ from each other on pairs of samples with different clinical diagnosis labels. The value of 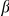 would therefore be the smallest of these counts. The value of 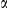 is chosen in a similar way, considering the features that do not differ from each other on samples with the same clinical diagnosis label, such that the combinatorial optimization problem also admits at least one feasible solution and this choice does not force a change on the value of 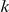 or 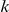. The 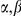 best features that explain the dichotomy between classes are chosen such that they not only satisfy the 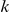 and 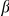 values, but also explain the differences and similarities of a greater number of pairs of samples. The determination of 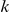 and selection of the best 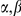 features that satisfy 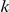 and 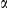 are done by solving the associated Integer Program (IP) using the ILOG CPLEX optimization package version 11.2. See [3], [6]–[11], [14], [16] for details on the IP formulations and other previous applications.

Next, classification is performed with 25 different classifiers of different types. Table 1 lists all the classifiers under consideration and their types. They are the same 24 classifiers that Gómez Ravetti and Moscato [3] considered plus the Bagging algorithm, which is considered one of the best classifiers [18] available in the Weka package [13]. Each classifier is run on the selected panel of features and meta-features, using the continuous (non-discretized) values, and all default parameters of Weka as of version 3.6.1 (The only exception is IBk, in which the parameter k is set to 2, to distinguish it from IB1. PAM's [19] threshold is also set to zero, to avoid the shrinkage process, which is also a feature selection procedure that we wish to avoid, as a more sophisticated method is already previously applied.) No fine tuning is done. Since an independent test set is already provided and the average performance of all classifiers is already considered, no cross-validation is performed.

**Table 1:** List of classifiers and their types.

Type	Classifier	Type	Classifier
bayes	BayesNet	bayes	NaiveBayes
bayes	NaiveBayesSimple	bayes	NaiveBayesUpdateable
functions	Logistic	functions	MultilayerPerceptron
functions	SimpleLogistic	functions	SMO
lazy	IB1	lazy	IBk
lazy	KStar	lazy	LWL
meta	AdaBoostM1	meta	Bagging
meta	Decorate	meta	RandomCommittee
meta	OrdinalClassClassifier	meta	MultiClassClassifier
meta	ClassificationViaRegression	rules	PART
trees	J48	trees	LMT
trees	NBTree	trees	RandomForest
other	PAM		

List of classifiers considered in this work and their types as categorised in Weka [13] version 3.6.1. No fine tuning is done, and all default parameters, as of the same version of the software, are used, except for IBk, in which k is set to two, to distinguish it from IB1. In PAM the threshold is set to zero to avoid the shrinkage process and force the classifier to use all the previously selected features. Please note that PAM [19] is not included in the Weka package.

The case-by-case analysis is done by plotting the histogram of the number of classifiers that disagree with the clinical diagnosis label given to each sample. With the objective of providing the classifiers only with training examples that characterise their class well, which would provide them with better hints for pattern searching, the samples for which more than 30% of the classifiers do not agree with the clinical diagnosis are removed from the training set. The process is then repeated with this (new) reduced training set until no more than than 30% of the classifiers disagree with the clinical diagnosis of all samples, the reduced training set yields the same signature as in the previous iteration, or the number of available training samples gets too low. The methodology of reducing the size of the training set by excluding samples, both in semi-supervised or unsupervised settings, is called “data pruning” and has been previously used to avoid overfitting and improve generalisation [20], [21].

## Results

The first fact worth mentioning is that, considering the expanded dataset, 118 out of the 120 proteins passed the entropy filter as part of a meta-feature, while only 12 of them pass when considered alone in the original dataset. More interestingly, 91 out of these 118 features passed the entropy filter only in metafeatures that do not include any of the 12 features that passed the entropy filter alone. In other words, these features are only interesting to distinguish between cases and controls when the imbalance information is considered, and their importance is not dominated by any of the features that are already known to be interesting.

Tables 2, 3, 4 compare the average results of three signatures obtained in this work and the two signatures previously identified known ones by Gómez Ravetti and Moscato [3] and Ray et al. [1] when performing on the Training Set, Test Set with NDC and samples with AD (referred simply as Test Set), and Test Set with MCI samples that developed and did not develop AD (Test Set MCI), respectively. The values shown are the average results of the 25 classifiers under consideration.

**Table 2:** Average results of classification performed on the Training Set.

*Training Set*						
Signature	avg acc	stdev acc	avg sens	stdev sens	avg spec	stdev spec
**S1**	0.944	0.045	0.952	0.038	0.936	0.065
**S2**	**0.980**	0.028	**0.980**	0.033	**0.981**	0.034
**S3**	0.974	0.032	0.974	0.039	0.975	0.035
**Ray et al.**	0.930	0.050	0.940	0.046	0.920	0.072
**Gómez Ravetti and Moscato**	0.902	0.057	0.900	0.061	0.903	0.070

The signature obtained by just selecting features that best complement Gómez Ravetti and Moscato's [3], S1, improves Ray et al.'s [1] classification accuracy on the training set of 93% to 94.4%, with improvements both in sensitivity and specificity. This signature includes the following features and meta-features: EGF, IL-1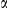, IL-3, TNF-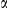, G-CSF, “BLC-RANTES”, “MIP-1d-IL-11”, “TNF-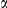 - ANG-2”, “TNF-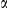 - FAS” and “IL-11-I-TAC”. The performance of the second signature, S2, obtained in the same manner after discarding samples s3, s7, s47, s66 and s77 is still better than that of the other signatures, with an even greater gap, reaching an average of 98% of learning accuracy. Even though this does not necessarily imply an improvement also on independent test sets, this provides good evidence that the discarded samples were indeed problematic. This signature includes the following features and meta-features: EGF, IL-1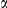, TNF-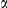, G-CSF, “EGF-IGFBP-2”, “GM-CSF-IL-1
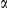
”, “IL-1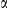-IL-11”, “MIP-1d-NT-3”, “PDGF-BB-VEGF-B” and “TNF-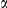-ANG-2”. The signature obtained by just discarding the single features from S2, S3, also shows a very good performance on the training set. It is remarkable that the average results differed by less than 1% from those from the 4 feature and 6 meta-feature signature. That suggests that the single features were not playing a key role to distinguish between AD and NDC, and supports the theory that there is useful information provided by the meta-features to distinguish between the classes.

**Table 3:** Average results of classification performed on the Test Set with NDC and samples that developed AD.

*Test Set*						
Signature	avg acc	stdev acc	avg sens	stdev sens	avg spec	stdev spec
**S1**	0.854	0.039	0.915	0.047	0.803	0.069
**S2**	0.905	0.031	0.937	0.047	0.878	0.040
**S3**	0.890	0.021	0.923	0.044	0.862	0.038
**Ray et al.**	0.906	0.030	0.917	0.042	0.896	0.051
**Gómez Ravetti and Moscato**	**0.923**	0.031	**0.948**	0.037	**0.902**	0.053

When performing on the independent test set with NDC and AD samples, Gómez Ravetti and Moscato [3] still hold the best results, obtaining an average of 92.3% of accuracy. Th e signature obtained by just selecting features to complement Gómez Ravetti and Moscato's [3] signature, S1, almost matched Ray et al.'s [1] sensitivity, even though it did not perform so well in terms of specificity. It includes EGF, IL-1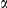, IL-3, TNF-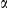, G-CSF, “BLC-RANTES”, “MIP-1d-IL-11”, “TNF-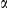 - ANG-2”, “TNF-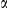 - FAS” and “IL-11-I-TAC”. The second signature, obtained in the same manner after discarding samples, performed significantly better, almost matching Ray et al.'s in accuracy. It includes EGF, IL-1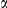, TNF-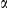, G-CSF, “EGF-IGFBP-2”, “GM-CSF-IL-1
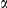
”, “IL-1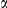-IL-11”, “MIP-1d-NT-3”, “PDGF-BB-VEGF-B” and “TNF-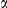-ANG-2”. Interestingly, the last signature, S3, obtained by just discarding the single features of S2, yields very similar results. That supports the theory that the (single) features were not playing a key role in distinguishing between AD and NDC on S2, and that the meta-features indeed hold useful information for that purpose.

**Table 4:** Average results of classification performed on the Test Set with samples that already had a MCI that developed AD or not.

*Test Set MCI*						
Signature	avg acc	stdev acc	avg sens	stdev sens	avg spec	stdev spec
**S1**	**0.704**	0.058	0.820	0.097	**0.602**	0.134
**S2**	0.678	0.033	0.818	0.066	0.555	0.071
**S3**	0.677	0.029	**0.840**	0.070	0.534	0.076
**Ray et al.**	0.662	0.046	0.755	0.112	0.581	0.081
**Gómez Ravetti and Moscato**	0.650	0.053	0.731	0.131	0.579	0.085

All the obtained signatures perform better than the previously know ones on the blinded test set with MCI samples that developed AD or not. Interestingly, the Ray et al.'s [1] sensitivity of 75.5% was improved to 84% on the signature obtained by discarding the single features of the signature obtained by selecting features that complement the part of Gómez Ravetti and Moscato's [3] signature that passed the entropy filter after discarding samples s3, s7, s47, s66 and s77. This signature is composed by “EGF-IGFBP-2”, “IL-1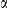-GM-CSF”, “IL-1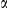-IL-11”, “MIP-1d-NT-3”, “PDGF-BB-VEGF-B” and “TNF-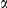-ANG-2”. As expected, since we did not have training samples that already had a MCI, the specificity was not improved.

### First Iteration of the Method

The first signature, obtained in the first iteration of the method depicted in the previous section, included the following features and meta-features: EGF, IL-1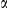, IL-3, TNF-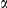, G-CSF, “BLC (chemokine (C-X-C motif) ligand 13) –RANTES (chemokine (C-C motif) ligand 5)”, “MIP-1d (chemokine (C-C motif) ligand 15) – IL-11 (interleukin 11)”, “TNF-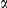
–ANG-2 (angiopoietin-2)”, “TNF-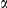
–FAS (Tumor Necrosis Factor receptor superfamily, member 6)” and “IL-11–I-TAC (chemokine (C-X-C motif) ligand 11)”. Referred as “S1”, it improved Ray et al.'s [1] classification accuracy of 93% to 94.4%. Gómez Ravetti and Moscato's [3] signature still holds the best results when performing on the test set with NDC and samples that developed AD, with 92.3% of accuracy in average. Their results still outperform all others both in sensitivity (94.8%) and specificity (90.2%).

When performing on the test set with MCI samples that developed AD and MCI samples that did not, this signature improved Ray et al.'s [1] accuracy of 66.2% to 70.4% mainly due to an improvement of 6.5% in sensitivity, which reached 82%. Although an improvement in specificity was not expected, since there are no MCI samples in the training set, it was also raised to 60.2%.

**Figure 6:**
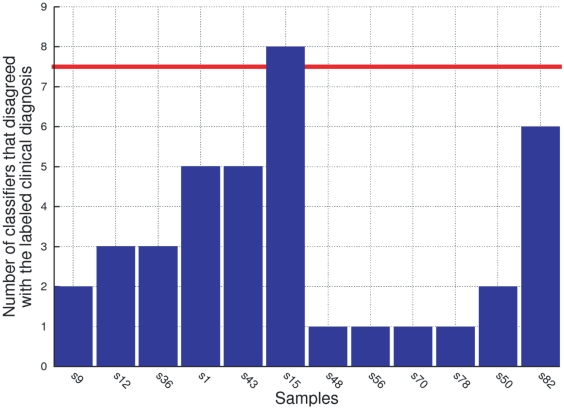
Histogram of the number of classifiers, out of the 25 under consideration, that disagreed with the clinical diagnosis attributed to the samples, when performing on the training set using the proposed 4 feature and 6 meta-feature signature. The performance of the signature obtained after the removal of samples s4, s7, s47, s66 and s77, against the training set, improved significantly. More than 30% of the classifiers disagreed with the clinical diagnosis of only one sample s15. However, since only 8 classifiers got it wrong, which was the threshold limit rounded up, and no classifier disagreed with the clinical diagnosis of this sample on the previous iteration, it was not considered problematic and the training set was not reduced even more. The signature used for this experiment includes the following features and meta-features: EGF, IL-1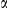, TNF-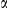, G-CSF, “EGF-IGFBP-2”, “GM-CSF-IL-1
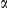
”, “IL-1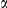-IL-11”, “MIP-1d-NT-3”, “PDGF-BB-VEGF-B” and “TNF-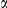-ANG-2”.

Figure 6 shows the number of classifiers that disagree with the clinical diagnosis label for each sample, when performing against the training set. It is interesting to notice that there is a set of samples that several classifiers, of a wide-range of different types, consistently disagree with the clinical diagnosis label attributed to a sample. Therefore, it is reasonable to think that these samples might either be mislabelled, that the clinical diagnosis is inadequate, or other latent clinical factors, such as the presence of other existing patient conditions (diseases, medication, or other factors) affected the cell signalling proteins present in this signature. This is consistent with Ray et al.'s [1] remark that there might be mislabelled samples in the dataset, due to the fact that the patients were still alive and an accurate diagnosis could only be issued with the post-mortem analysis of the brain cortex.

### Second Iteration of the Method

Following the hypothesis that some of the training samples might not have the correct label or might not characterise their target classes well, a new training set was generated by disconsidering samples for which more than 30% of the classifiers disagreed about the label: s3, s7, s47, s66 and s77. This threshold was determined through the visual analysis of Figure 6, aiming to cut off the highest histogram peaks while observing the samples that did not cluster to their clinical diagnosis label group also in Figure 4 (samples s47, s77, s54, s50, s66, s68, s3 and s1), and trying not to discard too many samples. It is interesting to notice that amongst the samples that did not cluster well on Figure 4 only sample s54 did not appear on Figure 6.

Using this new training set, a new signature was obtained on a second iteration of the proposed method. The new signature, referred as “S2” included the following features and meta-features: EGF, IL-1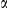, TNF-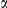, G-CSF, “EGF–IGFBP-2 (insulin-like growth factor binding protein 2, 36 kDa)”, “GM-CSF (colony stimulating factor 2 (granulocyte-macrophage)) –IL-1
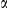
”, “IL-1
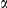
–IL-11”, “MIP-1d–NT-3 (neurotrophin 3)”, “PDGF-BB (platelet-derived growth factor beta polypeptide (simian sarcoma viral (v-sis) oncogene homologue)) –VEGF-B (vascular endothelial growth factor B)” and “TNF-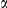
–ANG-2”. It is worth noticing that, after filtering the training set, the obtained signature no longer included IL-3 because the entropy filter discarded it. That could be because most of the information provided by IL-3 that distinguished cases and controls was in the excluded samples. Even though some of the meta-features were also replaced in this signature, that was not because they did not pass the entropy filter, but because the feature selection method chose to select different ones.

The performance of this second signature on the training set still outperforms the other signatures, reaching an average of 98% of learning accuracy, against Ray et al.'s [1] 93%. This second signature matches Ray et al.'s [1] accuracy of 90% against the Test Set with AD and NDC samples, but is still outperformed by Gómez Ravetti and Moscato's [3] accuracy of 92.3%.

The most remarkable characteristic of this new signature, however, is not the improvement in total accuracy, but in sensitivity, when the Test Set with samples that already had a MCI is used as benchmark.

**Figure 7:**
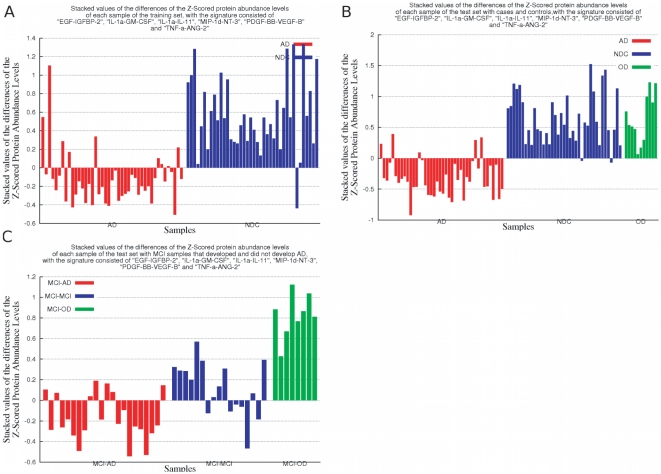
Stacked values of the differences of Z-Scores of only the meta-features of the 4 feature and 6 meta-feature signature. The signature used in this experiment includes the following meta-features: “EGF-IGFBP-2”, “IL-1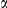-GM-CSF”, “IL-1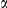-IL-11”, “MIP-1d-NT-3”, “PDGF-BB-VEGF-B” and “TNF-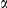-ANG-2”. Figures 7A–C present the stacked values of of the differences of Z-Scores in the training, independent test set of samples with AD and NDC samples, and the independent test set with MCI samples that developed and did not develop AD, respectively. The figures show that the stacked values of this panel of 6 meta-features are lower in those patients that will develop clinical symptoms of AD in two to five years. In Figures 7A–C the samples marked in red are those that developed AD, the samples marked in blue are those that did not develop AD (NDC Figures 7A–B, and samples that had a MCI and did not develop AD on the Figure 7C) and the ones marked in green are OD. Since these values correspond to a difference of Z-Scored values, the average is common and cancelled, thus the measured values of each protein are no longer relative to the variation of the other 119, but only of the other protein involved in the meta-feature. It is interesting to notice that this signature, composed only of meta-features, distinguishes well between AD and NDC on both training (see Figure 7A) and test with AD and NDC samples (see Figure 7B) sets and also on the test set with MCI samples that developed and did not develop AD (see Figure 7C). As shown on Figures 7B–C, it is also remarkable that the signature also distinguishes fairly well between samples that developed AD and OD.

Even though a loss in specificity is observed, a good performance for this item is not expected, as no training samples had MCI. Figure 7 shows that, using the second signature, more than 30% of the classifiers disagree about the clinical diagnosis label of only 1 sample (s15). Therefore a third iteration takes place with a new training set that disconsiders this sample. However, the obtained signature is the same as that obtained in the previous iteration, which interrupts the procedure.

### A Step Further

Finally, a signature consisted of the six meta-features of the previous signature is taken under consideration to evaluate their contribution on the observed performance. The meta feature “GM-CSF–IL-1
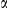
” was replaced by its equivalent meta feature “IL-1
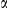
–GM-CSF”. They are equivalent from the feature selection point of view because they have the same absolute value. In other words, the 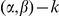 Feature Set feature selection approach could select either meta-feature and discard the other, as it would be redundant. Therefore only one is present on the dataset, as this halves the size of the associated problem. This modification is proposed only to help with the visualisation on Figure 5.

Figures 5A–C present the stacked values of of the differences of Z-Scores in the training, independent test set of samples with AD and NDC samples, and the independent test set with MCI samples that developed and did not develop AD, respectively. The figures show that the stacked values of this panel of 6 meta-features are lower in those patients that will develop clinical symptoms of AD in two to five years.

It is interesting to notice that this signature, composed only of meta-features, distinguishes well between AD and NDC on both training (see Figure 5A) and test with AD and NDC samples (see Figure 5B) sets and also on the test set with MCI samples that developed and did not develop AD (see Figure 5C). As shown on Figures 5B–C, it is also remarkable that the signature also distinguishes well between samples that developed AD and OD.

The fact that the average results of this set of tables differed from those of the previous signature by less than 1% on the training set, 2% on the Test Set and even yielded a better sensitivity on the Test Set MCI suggests that these particular single features were not playing a key role to distinguish between AD and NDC in this signature, and supports the theory that there is useful information within the meta-features to distinguish between the classes. Also, since the results for Test Set MCI also did not change significantly, it is reasonable to say that the sample pruning and usage of metafeatures introduced a good generalization in the signature.

## Discussion

ANG-2 (ANGPT2, Angiopoietin 2) is a regulator of angiogenesis. Ahmed et al. [22] have recently shown that apoE(−/−) mice that were fed a Western diet had a significant reduction of atherosclerotic lesion size and oxidized LDL and macrophage content of the plaques after a single systemic administration of ANG-2 adenovirus [22]. Thirumangalakudi et al. observed that ANG-2 levels in microvessels were increased in AD patients but not in age-matched controls [23].

Neurons and neuronal nuclei in hippocampus have been reported to express RANTES (CCL5, chemokine (C-C motif) ligand 5) which could induce an inflammatory cell infiltration in AD [24] (see also [25]). RANTES has also been observed as upregulated in the cerebral microcirculation of AD patients (in another study by members of the same team [26]), as well as by other groups of researchers [25], [27]. Even though this biomarker does not appear in any of the selected meta-features of the 10 feature signature, it appeared quite intensely in the 290 feature signature of Figure 4.

FAS/CD95, the Tumor Necrosis Factor Receptor Super Family 6 gene (TNFRSF6), has also appeared in our signature. Increased levels in cerebrospinal fluid in AD patients have been reported in [28]. The upregulation has been motivating several disease mechanistic explanations [29]–[37]. Several researchers have then tried to find polymorphisms that may have been correlated with AD and that avenue of research has not been highly prosperous [38]–[42], but there exist some studies with relatively positive results [34], [41], [42].

Serum levels of BLC have been reported as being elevated in multiple sclerosis [43]. Weiss et al. have shown that neural precursors cells express a receptor for BLC [44]. Upregulation of BLC was observed in scrapie-infected brain tissue in [45]. Baker, Martin and Manuelidis also reported in 2002 that microglia of Creutzfeldt-Jakob disease-infected brains characteristically present an upregulation of BLC [46]. In contrast, the selection of a metafeature involving RANTES and BLC indicates that the difference of z-scores of RANTES and BLC are differentially observed in AD and NDC participants of this study. As RANTES upregulation in AD has been put forward as a mechanism for neuroprotection [26] the concurrent lack of upregulation of BLC may point to a protective response that is not properly functioning in early AD worth investigating.

NT-3 (neurotrophin 3, Nerve growth factor 2) [47]–[50] also appears in a meta feature with MIP-1delta (CCL15, chemokine (C-C motif) ligand 15). This is a novel biomarker that may interest several AD researchers as the selective targeting of several neurotrophin receptors has been proposed as a viable mechanism of intervention for neuroprotection [51]–[56] (with much of the attention being on the p75(NTR), the common neurotrophin receptor [53], [57]–[66]). Hippocampal upregulation of NT-3 has been observed in mouse models of AD [67]. The ratio of NGF/NT-3 (NGF is the Nerve growth factor) was observed to be significantly upregulated in AD (in a comparison with control samples) in hippocampus and frontal cortex [68]. Lesne et al. propose that NT-3 reduces Abeta-induced apoptosis by limiting the cleavage of caspase-3, caspase-8, and caspase-9 [69].

The joint identification of PDGFB/PDGF-BB (platelet-derived growth factor beta polypeptide (simian sarcoma viral (v-sis) oncogene homologue), a member of the neurotrophic factor family [70] and VEGFB/VEGF-B (vascular endothelial growth factor B) is intriguing. At the time of the publication of Ray et al.'s [1] manuscript, on which database our work is based, VEGF-B was generally recognized as an angiogenic factor, although of low activity. Almost a year later, Poesen et al. proposed that the 60 kDa VEGF-B isoform is a neuroprotective factor [71] and Falk et al. later shown that exogenous VEGF-B is neuroprotective in a culture model of Parkinson's disease [72]. New roles for VEGF-B are being discovered, like those on lipid uptake, more specifically, on controlled endothelial uptake of fatty acids [73].

A link with CSF2/GM-CSF (colony stimulating factor 2 (granulocyte-macrophage)) with AD is more well-established [36], [74]–[78]. A similar remark could be applied to IGFBP-2 [79], [80].

It is also interesting to note that TNF-alpha was present in all signatures. A very recent study by O'Bryant et al. with serum protein based multiplex biomarker data from 197 patients diagnosed with AD and 203 controls showed a 0.74 fold change in AD patients [81]. They have also observed a 0.7 fold change on G-CSF (colony stimulating factor 3 (granulocyte)). These results may somewhat indicate that two of the proteins in Gómez Ravetti and Moscato's 5-protein signature [3], and the signature obtained on the first iteration of our method may indeed change in both studies.

The work of [82] showed that there are indications that plasma levels of EGF are linked with cognitive decline in Parkinsons disease, indicating it may not be entirely AD-specific as single biomarker.

### Conclusions

In this paper we modelled the relative protein imbalance using “artificial” features, called “metafeatures”. Selecting features and metafeatures using the 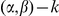 Feature Set Problem approach it was possible to show that a specific pattern of cell signalling imbalance in blood plasma provided valuable information for distinguishing between NDC and AD patients. Moreover, the obtained signatures were able to predict AD in patients that already had MCI with up to 84% sensitivity, while also maintaining a strong prediction accuracy of 90% on a independent dataset with NDC and AD samples.

Using a data-pruning strategy, we found good evidence that, as already remarked by Ray et al. [1], the dataset indeed had “suspicious” training samples, that could have the wrong diagnosis label or did not characterise their classes well due to other clinical factors. We believe that their removal could have introduced better generalisation to the obtained signatures. That also supports the theory that, even though our reported accuracy for predicting AD and NDC is lower than the best reported [3], it does not necessarilly mean that the signature does not perform well, as there might also be test samples with the wrong clinical diagnosis or that also do not characterise their classes well due to other clinical factors.

The novel biomarkers uncovered with the proposed method now confirms ANG-2, IL-11, PDGF-BB, CCL15/MIP-1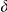; and supports the joint measurement of other signalling proteins in plasma not previously discussed: GM-CSF, NT-3, IGFBP-2 and VEGF-B.
